# Molecular and Morphological Analysis Reveals Five New Species of *Zygophiala* Associated with Flyspeck Signs on Plant Hosts from China

**DOI:** 10.1371/journal.pone.0110717

**Published:** 2014-10-20

**Authors:** Liu Gao, Mian Zhang, Wanyu Zhao, Lu Hao, Hongcai Chen, Rong Zhang, Jean C. Batzer, Mark L. Gleason, Guangyu Sun

**Affiliations:** 1 Department of State Key Laboratory of Crop Stress Biology in Arid Areas and College of Plant Protection, Northwest A&F University, Yangling, Shaanxi Province, China; 2 Department of Plant Pathology and Microbiology, Iowa State University, Ames, Iowa State, United States of America; California Department of Public Health, United States of America

## Abstract

Species in the genus *Zygophiala* are associated with sooty blotch and flyspeck disease on a wide range of hosts. In this study, 63 *Zygophiala* isolates collected from flyspeck colonies on a range of plants from several regions of China were used for phylogeny, host range and geographic distribution analysis. Phylogenetic trees were constructed on four genes - internal transcribed spacer (ITS), partial translation elongation factor 1-alpha (TEF), β-tubulin (TUB2), and actin (ACT) – both individually and in combination. Isolates were grouped into 11 clades among which five new species, *Z. emperorae, Z. trispora, Z. musae, Z. inaequalis* and *Z. longispora,* were described. Species of *Zygophiala* differed in observed host range and geographic distribution. *Z. wisconsinensis* and *Z. emperorae* were the most prevalent throughout the sampled regions of China, whereas *Z. trispora, Z. musae, Z. inaequalis* and *Z. longispora* were collected only in southern China. The hosts of *Z. wisconsinensis* and *Z. emperorae* were mainly in the family Rosaceae whereas *Z. trispora, Z. musae, Z. inaequalis* and *Z. longispora* were found mainly on banana (*Musa* spp.). Cross inoculation tests provided evidence of host specificity among SBFS species.

## Introduction

Sooty blotch and flyspeck (SBFS) is a late-season disease whose component fungi form colonies on the waxy epicuticles of a wide range of hosts such as apples, pears, carnations and bananas [1]–[5]. Fungi in the SBFS complex cause blotching that reduces the sale value of fruits and other crops [6]. This disease was initially thought to be caused by a single species, *Dothidea pomigena* [7]. Colby [2] later separated the disease complex into two types of signs according to colonies on host surface, each with a single causal species. Colonies with dark mycelial mats were denoted as sooty blotch and were ascribed to *Gloeodes pomigena*, whereas clusters of shiny, black, round to ovoid, sclerotium-like bodies with no visible mycelial mat were denoted as flyspeck and were ascribed to *Leptothyrium pomi* [8]. The pathogens of latter type were reclassified as several species of *Zygophiala* [9]. Currently, several intermediate mycelial types of SBFS are associated with this complex. Those with dark mycelial mats were separated into ramose, punctate, fuliginous, ridged honeycomb and fleck, and those with no visible mycelial mat were classified as flyspeck, discrete speck, and compact speck [1].

The SBFS fungal complex is now recognized to be highly diverse, including dozens of species in more than 10 genera [3]. As additional surveys have been conducted, more SBFS fungi have been discovered [10]–[11]. The genus *Zygophiala*, which causes flyspeck sign on the host [3], [9], is among the most important and widespread groups of SBFS fungi, and has received increasing research attention in recent years [9], [12]–[13].

The genus *Zygophiala* was established in 1945 [15] and the type species of the genus, *Z. jamaicensis*, was initially described as the causal agent of banana leaf speckle in Jamaica. It was subsequently presumed that this anamorph species (*Z. jamaicensis*) was also the cause of flyspeck on apple [16], which was previously described as the teleomorph species *Schizothyrium pomi* [8]. Although evidence for the anamorph-teleomorph connection was not presented, the assumption was widely accepted in subsequent literatures [4], [17]–[18], and *Z. jamaicensis* was thought to be the only species in the genus [8], [16]–[18] despite observation of considerable variations in cultural characterizations [16], [19]. Batzer et al. [9] described four species of *Zygophiala* on apple that included the anamorph of *S. pomi* and three new species, *Z. cryptogama*, *Z. tardicrescens* and *Z. wisconsinensis*. Furthermore, as the conidial size of the anamorph of *S. pomi* measured in their study differed from that previously reported for *Z. jamaicensis*, Batzer and co-workers concluded that *S. pomi* was not the teleomorph of *Z. jamaicensis* [9].

Recently, more *Zygophiala* species have been discovered. In China, two additional species, *Z. qianensis* [14] and *Z. cylindrica* [13] were set up from apple. In addition, *Z. wisconsinensis* and *Z. cryptogama*, which were first described in the USA, were also reported in China [13], [20]. From 2009 to 2012, a survey of flyspeck colonies was conducted in several provinces throughout China on many plant hosts. The aim of this paper was to identify the species assemblage, host range and geographic distribution of *Zygophiala* in China and describe unknown species based on morphological characteristics and DNA phylogeny.

## Materials and Methods

### Isolates

Subsections of flyspeck colonies were transferred from host plants to potato dextrose agar (PDA) [21]. Representative isolates were deposited in the China General Microbiological Culture Collection Center (CGMCC) (Beijing, China) and dried cultures were deposited at the Herbarium Mycologicum Academiae Sinicae (HMAS) (Beijing, China). Host tissues exhibiting flyspeck colonies were excised and pressed between paper towels until dry and deposited in the Fungal Laboratory of Northwest A&F University. All isolates were purified and stored in glycerol at −80°C in the same laboratory [1], [11]. Nomenclatural novelties and descriptions were deposited in MycoBank (www.MycoBank.org) [22]. The isolate coda, locations, hosts and GenBank numbers used in the study are shown in Table S1.

### DNA extraction, polymerase chain reaction and sequencing

Amplification and sequencing of isolates obtained in China were performed at Northwest A&F University and isolates from the USA were processed at Iowa State University. Template DNA for polymerase chain reaction (PCR) was obtained by scraping mycelium from single-conidium isolates that had been grown on PDA plates for 4 to 6 weeks. DNA was extracted from the mycelium according to the protocol of Li et al. [23]. The internal transcribed spacer region (ITS) of ribosomal DNA was amplified for all 63 isolates using primer pair ITS1-F/ITS4 [24]. A subset of 28 isolates, selected by the ITS sequence data analysis to represent the range of genetic diversity (Table S1), was further sequenced for partial translation elongation factor 1-alpha (TEF), partial actin (ACT) and β-tubulin (TUB2). The partial TEF, ACT, and TUB2 regions were amplified separately with primer pairs EF-728F/EF-986R [25], ACT512F/ACT783R [26] and Bt2a/Bt2b [27], respectively. For some TEF sequences that could not be amplified by primer EF-986R, primer EF-2 [28] was used as alternative reverse primer. The PCR amplification mixture consisted of 1 unit of Taq polymerase, 1× PCR buffer, 2 mM MgCl_2_, 0.2 mM of each dNTP, 0.4 µM of each primer, and 2 µL template DNA, and was made up to a total volume of 25 µL with sterile water. In China, reactions were performed on a BIO-RAD PCR System S1000TM Thermal Cycler; in Iowa, reactions were performed on a PTC-100 thermocycler (MJ Research Inc., Waltham, Massachusetts). In both locations cycling conditions were an initial denaturation at 94°C for 95 s followed by 35 cycles of denaturation at 94°C for 35 s, annealing at 52°C for ITS for 60 s, extension at 72°C for 2 min, and a final 10-min extension step at 72°C. The cycling parameters for TEF consisted of a denaturation step at 94°C for 5 min, followed by 34 cycles at 94°C for 45 s, 55°C for 30 s, 72°C for 45 s and a final cycle at 72°C for 10 min. The cycling parameters for partial ACT and TUB2 regions consisted of a 3-min denaturing step at 94°C followed by 34 cycles at 94°C for 45 s, 57°C for ACT and 50°C for TUB2 for 1 min, 72°C for 1 min and a final cycle of 10 min at 72°C. Purifying and automated sequencing of the PCR product was performed at Organism Technology, Shanghai, China with forward and reverse primers in two directions, respectively. In Iowa purifications and sequencing were conducted as described in Batzer et al. [1].

### Sequence alignment and phylogenetic analysis

**Figure 1:**
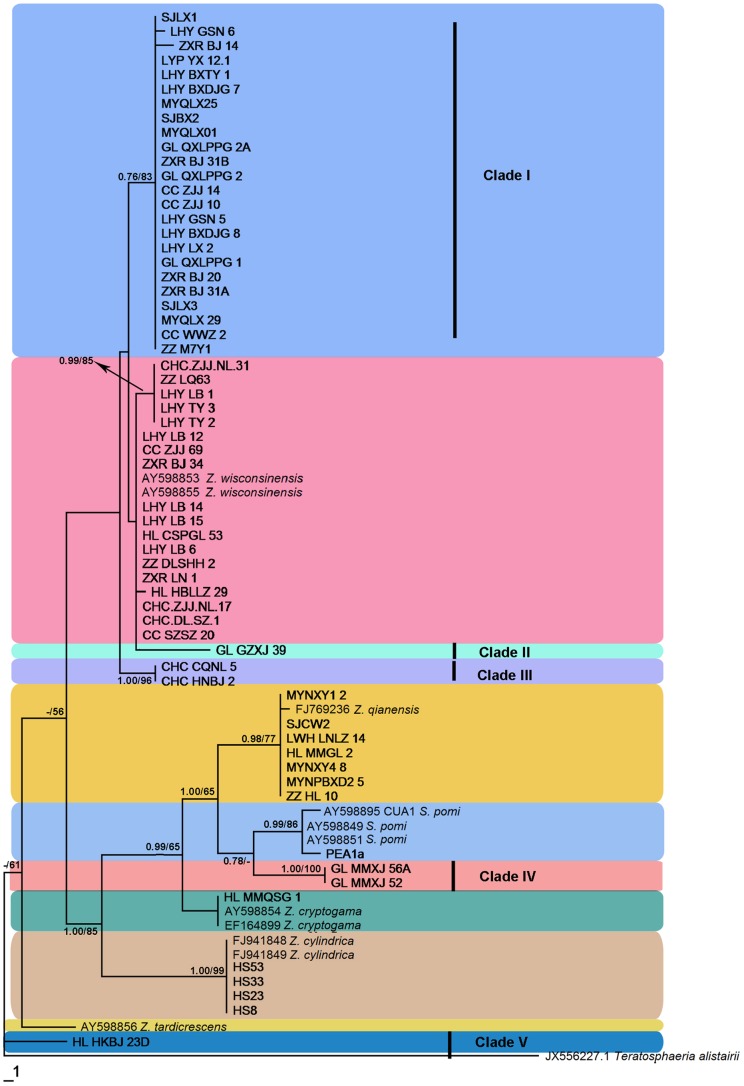
Phylogram of the Maximum Parsimony tree revealed by PAUP v.4.0b10. Bayesian tress showed the same topology as revealed by MP tree. Bayesian posterior probabilities (before the/) above 0.5 and parsimony bootstrap proportions (after the/) higher than 50% and were indicated along branches determined from ITS sequences. The tree is rooted to *Teratosphaeria alistairii* and different species are covered different colours. Sequences generated in this study were shown in bold. The holotype for *Z. jamaicensis* was not available for sequencing.

**Figure 2:**
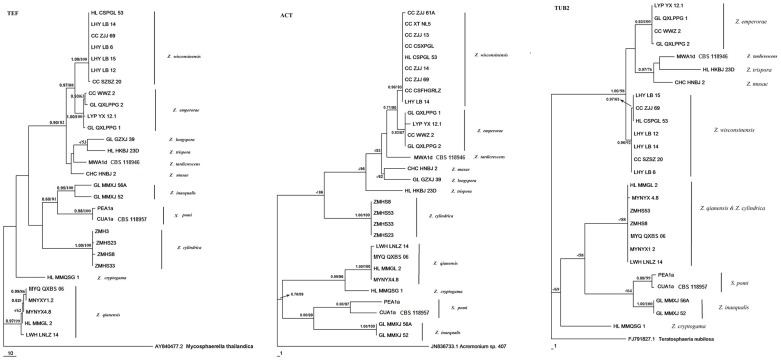
Phylogram of the Maximum Parsimony tree. Trees of elongation factor 1-alpha (TEF), partial actin (ACT) and β-tubulin (TUB2) showing phylogenetic placement of different species of Zygophiala. Numbers at branching nodes represent Bayesian Posterior probabilities above 0.5/bootstrap values higher than 50% (1000 replicates).

**Figure 3:**
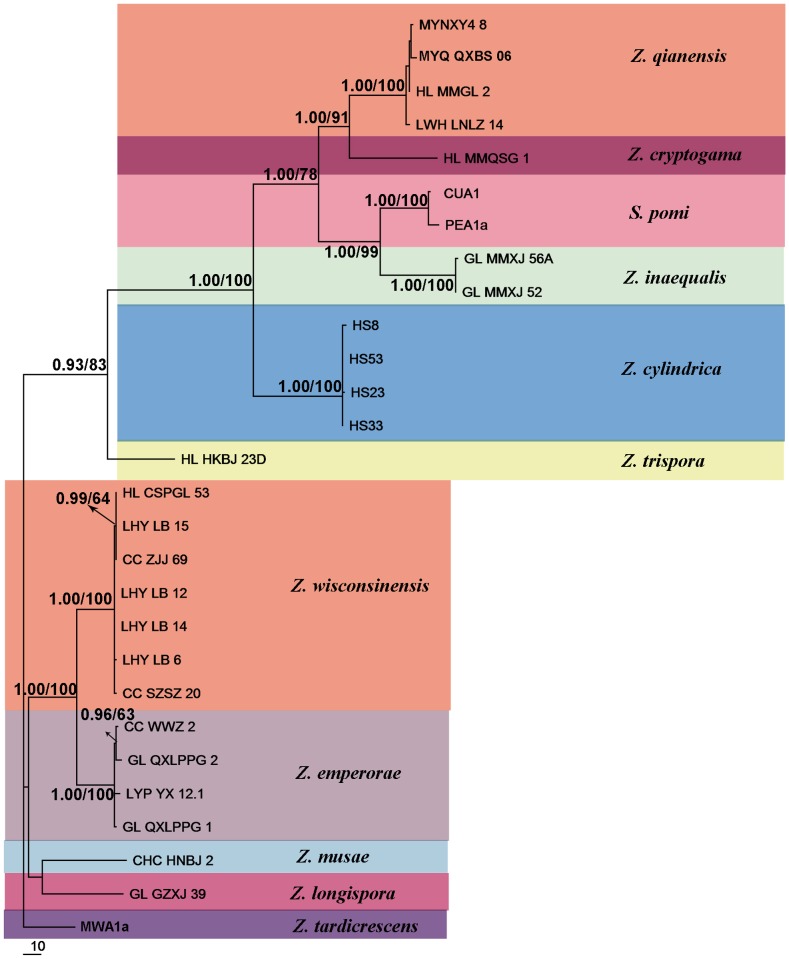
Maximum Parsimony tree derived from analysis of combined dataset (partial TEF, ACT and TUB2). Branches having Bayesian posterior probabilities >0.5 and bootstrap values >50% are indicated at nodes as BPP/MP.

The ITS sequences generated in this study, along with other *Zygophiala* spp. and outgroup sequences downloaded from NCBI's GenBank sequence database (Table S1), were imported into BioEdit [29] to be compared and analyzed. Preliminary alignments of the multiple sequences were conducted using CLUSTAL X [30], with manual adjustment using BioEdit for visual improvement where necessary. Isolates with redundant ITS sequences obtained from the same orchard were eliminated from the dataset and eventually 63 isolates were analyzed. Of these, 29 were used for TEF, TUB2 and ACT analysis and ITS-TEF-ACT-TUB2 matrix analysis. Newly generated sequences were deposited in GenBank (Table S1). Alignments and the representative trees (Figure 1, Figure 2 and Figure 3) were deposited in TreeBASE (http://purl.org/phylo/treebase/phylows/study/TB2:S15750).

Maximum parsimony (MP) analysis was performed with PAUP v.4.0b10 [31]. Heuristic searches were conducted with 1000 random sequence additions and tree bisection-reconnection (TBR) branch swapping algorithms, collapsing zero-length branches, and saving all minimal length trees [32]. All characters were unordered and of equal weight and gaps were treated as missing data. Branches of zero length were collapsed and all multiple, equally parsimonious trees were saved. Measures calculated for parsimony included tree length, consistency index, retention index and rescaled consistency index (TL, CI, RI and RC, respectively). To assess the robustness of clades and internal branches, a strict consensus of the most parsimonious trees was generated and a bootstrap analysis of 1,000 replications was performed. The outgroups were *Teratosphaeria alistairii* in ITS analysis, *Mycosphaerella thailandica* in TEF analysis, *Acremonium* sp. in ACT analysis and *Teratosphaeria nubilosa* in TUB2 analysis. Sequences generated in this study were deposited in GenBank (Table S1).

Bayesian analysis was conducted on the same aligned dataset and the likelihood model parameters were calculated for the ITS gene regions. MrModeltest v. 2.2 [33]–[34] was used to determine the best nucleotide substitution model. Phylogenetic analyses were performed with Mrbayes 3.2.2 [35] applying a general time-reversible (GTR) substitution model with gamma (G) and proportion of invariable site (I) parameters to accommodate variable rates across sites. The Markov Chain Monte Carlo (MCMC) analysis of 4 chains with two runs started from random tree topology and lasted 5,000,000 generations. Trees were saved each 1000 generations, resulting in 10,002 saved trees. Burn-in was set at 1,250,000 generations when the likelihood values were stationary, leaving 7502 trees from which the consensus trees and posterior probabilities were calculated and analyzed. A majority rule consensus tree of all remaining trees was calculated.

Bayesian posterior probabilities (BPP) higher than 0.5 and MP bootstrap support (BS) above 50% are given in Fig. 1–3 at the first and second positions above or below the branches.

### Morphology of SBFS isolates

Colony descriptions were made after 2 wk of growth on oatmeal agar (OA) plates at 25°C in darkness. Measurements of fungal structures were made from cultures grown on OA. Wherever possible, 30 measurements were made of structures mounted in lactic acid, with the extremes of measurements given in parentheses. For conidial measurements, the 95% percentiles are presented and extremes given in brackets [9].

### Pathogenicity on apple

From isolates used in morphological characterization, one isolate of each species was selected for pathogenicity tests on apples, both in the field and in an incubator. Preparation of inoculum suspensions of isolates GL-QXLPPG-1, HL-HKBJ-23D, CHC-HNBJ-2, GL-MMXJ-52 and GL-GZXJ-39 (the latter four were initially isolated from *Musa basjoo*), provisionally representing five new species of *Zygophiala*, followed protocols of Batzer et al. [1]. Single-conidial isolates were grown on 2% potato-dextrose agar (PDA) for one month. Excess agar was cut away and the colony was transferred to three to four 1.5 ml plastic centrifuge tubes. Inoculum suspensions containing hyphae and conidia were blended with 600 µl sterile deionized water (SDW) and ground with a vortex oscillator (Model QL-901, Kylin-Bell Lab Instruments Company Limited, Jiangsu Province, China) for 1 min. After that, they were refrigerated until use, which was within 2 h of preparation.

Field-inoculation followed a method first described by Batzer et al. [1] and modified by Gao et al. [12]. Immature apples (5 to 6 cm diameter; cv. Fuji) on trees in an orchard in Yangling (N34°18′, E108°02′) were used for inoculation. Apples were surface-sterilized with 70% ethanol and then allowed to dry for 2 min. For each isolate, five fruits were swabbed with inoculum suspension using sterilized brushes; on each apple, three inoculated areas, each 1 to 2 cm in diameter, were marked. A total of five control fruits were treated with distilled water instead of the inoculum suspensions. After inoculation, fruits were covered with polyethylene bags (20×15 cm) for 10 wk before they were examined for flyspeck colonization. To allow for some aeration, each corner of a bag was removed by making a 1.5-cm-long diagonal cut using a scissors. Orchard experiments were conducted from early July through mid-Sep. 2013. No specific permissions were required for these locations and the field studies did not involve endangered or protected species. At harvest, the bags were removed and the apples were transferred to the laboratory. Fungi that displayed flyspeck colonies were photographed and re-isolated onto PDA, and DNA was extracted from mycelia as described in Materials and Methods. Colony morphology and ITS sequences were compared to those of each original isolate.

Inoculation in an incubator at Northwest A&F University followed the protocol described above for field inoculation. Mature inoculated apple fruit (cv. Gala) were transferred to an incubator with 12 h fluorescent light at 28°C and 85% relative humidity (RH) alternated with 12 h darkness at 25°C with 100% RH for 1 month, then examined for signs of flyspeck and photographed.

### Nomenclature

The electronic version of this article in Portable Document Format (PDF) in a work with an ISSN or ISBN will represent a published work according to the International Code of Nomenclature for algae, fungi, and plants, and hence the new names contained in the electronic publication of a *PLOS ONE* article are effectively published under that Code from the electronic edition alone, so there is no longer any need to provide printed copies. In addition, new names contained in this work have been submitted to MycoBank from where they will be made available to the Global Names Index. The unique MycoBank number can be resolved and the associated information viewed through any standard web browser by appending the MycoBank number contained in this publication to the prefix http://www.mycobank.org/MB/. The online version of this work is archived and available from the following digital repositories: PubMed Central, LOCKSS.

## Results

### Phylogenetic analysis

The number of characters, the substitution models used and other statistical values resulting from analysis of the respective datasets are presented in Table S2.

Maximum parsimony (MP) (Figure 1) and Bayesian analysis of the ITS dataset resulted in trees with similar topologies. Eleven clades were delimited, five of which (Clade I – Clade V) were newly recorded. In both trees, 24 isolates of Clade I formed a weakly supported lineage (BPP  = 0.76, BS  = 83%). ITS analyses gave low bootstrap support within Clades I, II, III and Clade *Zygophiala wisconsinensis* in both the Bayesian trees (BPP<0.5) and MP trees (BS<50%) although their morphologies were distinguishable. Clades IV and V each formed a distinct clade.

Detailed analysis was performed using three protein-coding genes, TEF, TUB2 and ACT. However, we did not obtain the TUB2 sequences of GL-GLXJ-39 that distinguished Clade II in ITS analysis and the TUB2 sequences of HS23 and HS33 that clustered with *Z. cylindrica*. These three genes showed substantially more variation among our study isolates than was present in the ribosomal sequences. MP and BI analyses of the separate protein-coding datasets resulted in trees with similar topologies in the main clades, with some variability among subclades (Figure 2). All gene sequences strongly grouped isolates included in Clade I in Fig. 1 and *Z. wisconsinensis* within different clades (with 88%, 90% and 98% support, respectively) (Figure 2). In the TUB2 tree, the 29 *Zygophiala* isolates were separated into 11 well-supported lineages, but *Z. cylindrica* and *Z. qianensis* fell into a single clade and could not separate.

*Z. tardicrescens* isolate MWA1a was used as the outgroup for the multi-gene ITS-TEF-ACT-TUB2 matrix analysis. The TUB2 sequences of GL-GZXJ-39, HS23, and HS33 were not included because we could not sequence them. The combined phylogenetic trees from MP and BI analysis had the same overall topology as the four separate gene regions (Figure 1 and Figure 2). The phylogenetic trees obtained from the combined dataset (Figure 3) showed 11 distinct, well supported lineages in the genus *Zygophiala*.

### Taxonomy

Sixty-one isolates obtained in this work and 12 isolates obtained from GenBank were divided into 11 groups, of which five groups were previously unreported based on their morphology on cultural media, growth characteristics and DNA phylogeny. Five new species of *Zygophiala* were distinguished and are described below.

**Figure 4:**
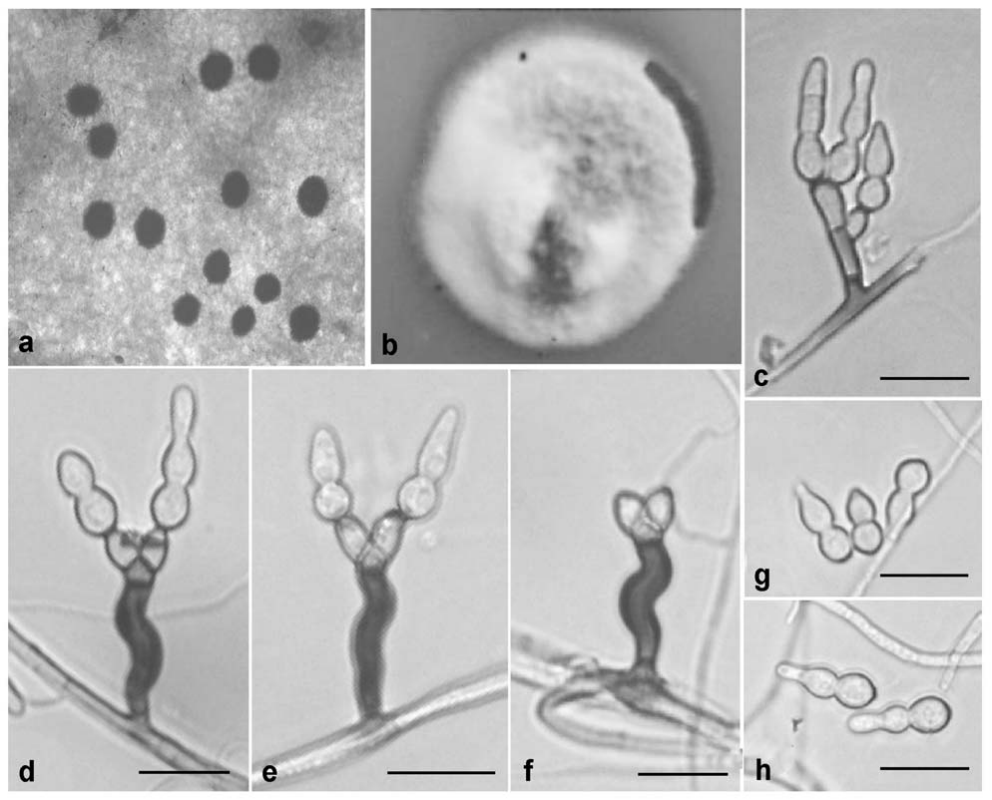
*Zygophiala emperorae* isolate GL-QXLPPG-1. (a) flyspeck signs on apple peel (b) 2-week-old colony on OA (c–e) conidiophores and conidia (f) conidiophores (g, h) conidia. bars  = 20 µm.

***Zygophiala emperorae.*** G.Y. Sun & Liu Gao, sp. nov. (Fig. 4)

[urn:lsid:indexfungorum.org:names: 808237]

#### Etymology

This species was first isolated from samples collected from Baoji City, Shaanxi Province, China. In ancient times, Emperors Yan and Huang built their tribes in the region around Baoji, and these tribes are often described as the ancestors of the Chinese people. We named this species in memory of our two ancestors.

*Conidiophores* arising from superficial hyphae, 2–3 µm wide, erect, scattered, 3-septate, subcylindrical, mostly irregular flexuous, consisting of a hyaline supporting cell that gives rise to a smooth, dark brown stipe, 16–27×4–6 µm (from basal septum to below phialide), terminating in a finely verruculose, medium brown apical cell, 3–6×4–6 µm, that gives rise to two (rarely one) medium brown, finely verruculose, doliiform to ellipsoidal, polyblastic conidiogenous cells, 4–8×4–6 µm, with 1–2 prominent scars, apical and lateral, darkened, thickened, somewhat refractive, 2 µm wide. *Conidia* solitary, fusiform to obclavate, hyaline, smooth and thick-walled, granular, transversely 0–2-septate, prominently constricted at septa, 1-septate (rarely median), (13–) 15–20 (–23) × (5–) 6–8 (–11) µm, 2-septate, 18–28×6–9 µm, apex obtuse, base subtruncate, with a darkened, thickened hilum, 2 µm wide.

#### Cultural characteristics

On oatmeal agar (OA), 2-week-old colonies spreading with aerial mycelium and smooth, regular margins, pale white. Diameter of 2-week growth at 25°C was 25 mm.

#### Holotypus

HMAS244991 ( =  GL-QXLPPG-1) (dried culture). From apple (*Malus × domestica* Borkh.) fruit with flyspeck, Qian County, Shaanxi, China, Oct. 2010, L. Gao. Ex-type culture CGMCC3.15250 ( =  GL-QXLPPG-1).

#### Additional isolates examined

China, Shaanxi, Qian County, from cuticle of apple (*M. × domestica* Borkh.) fruit with flyspeck, Oct. 2011, L. Gao, GLQXLPPG2A; China, Shaanxi, Qian County, from apple (*M. × domestica* Borkh.) fruit with flyspeck, Oct. 2011, L. Gao, GLQXLPPG2; China, Gansu, from apple (*M. × domestica* Borkh.) fruit with flyspeck, Oct. 2009, H. Y. Li., LHYGSN5; China, Gansu, from apple (*M. × domestica* Borkh.) fruit with flyspeck, Oct. 2009, H. Y. Li., LHYGSN6; China, Shaanxi, Xianyang, from apple (*M. × domestica* Borkh.) fruit with flyspeck, Oct. 2009, H. Y. Li, LHYBXTY1; China, Shaanxi, Xianyang, from apple (*M. × domestica* Borkh.) fruit with flyspeck, Oct. 2009, H. Y. Li, LHYBXDJG7; China, Shaanxi, Xianyang, from apple (*M. × domestica* Borkh.) fruit with flyspeck, Oct. 2009, H. Y. Li, LHYBXDJG8; China, Shaanxi, Baoji, from apple (*M. × domestica* Borkh.) fruit with flyspeck, Oct. 2009, H. Y. Li, LHYLX2; China, Shaanxi, Baoji, from apple (*M. × domestica* Borkh.) fruit with flyspeck, Sep. 2009, J. L. Zhuang, SJLX1; China, Shaanxi, Baoji, from apple (*M. × domestica* Borkh.) fruit with flyspeck, Sep. 2009, J. L. Zhuang, SJLX2; China, Shaanxi, Baoji, from apple (*M. × domestica* Borkh.) fruit with flyspeck, Sep. 2009, J. L. Zhuang, SJLX3; China, Shaanxi, Baoji, from apple (*M. × domestica* Borkh.) fruit with flyspeck, Oct. 2008, Y. Q. Ma, MYQLX01; China, Shaanxi, Baoji, from apple (*M. × domestica* Borkh.) fruit with flyspeck, Oct. 2008, Y. Q. Ma, MYQLX25; China, Shaanxi, Baoji, from apple (*M. × domestica* Borkh.) fruit with flyspeck, Oct. 2008, Y. Q. Ma, MYQLX29; China, Liaoning, from pear (*Pyrus bretschneideri* Rehd) fruit with flyspeck, Sep. 2010, C. Chen, CCWWZ2; China, Hunan, Zhangjiajie, from plum (*Prunus salicina* Lindl.) fruit with flyspeck, Oct. 2010, C. Chen, CCZJJ10; China, Hunan, Zhangjiajie, from plum (*Prunus salicina* Lindl.) fruit with flyspeck, Oct. 2010, C. Chen, CCZJJ14; China, Shaanxi, Baoji, from apple (*M. × domestica* Borkh.) fruit with flyspeck, Sep. 2007, X. R. Zhai, ZXRBJ14; China, Shaanxi, Baoji, from apple (*M. × domestica* Borkh.) fruit with flyspeck, Sep. 2007, X. R. Zhai, ZXRBJ20; China, Shaanxi, Baoji, from apple (*M. × domestica* Borkh.) fruit with flyspeck, Sep. 2007, X. R. Zhai, ZXRBJ31A; China, Shaanxi, Baoji, from apple (*M. × domestica* Borkh.) fruit with flyspeck, Sep. 2007, X. R. Zhai, ZXRBJ31B; China, Yunnan, Yuxi, from pawpaw (*Chaenomeles sinensis* (Thouin) Koehne) fruit with flyspeck, Oct. 2010, Y. P. Lei, LYPYX12.1; China, Shaanxi, Baoji, from apple (*M. × domestica* Borkh.) fruit with flyspeck, Sep. 2007, Z. Zhang, ZZM7Y1.

#### Notes

Morphologically, *Z. emperorae* is similar to *Z. qianensis* [14] (Table S2). The two species can be distinguished easily by growth rate. In culture, *Z. emperorae* grows relatively rapidly, reaching 25vmm diam on OA after 2 wk at 25°C, whereas *Z. qianensis* reached only 11 mm after 2 wk. Also, conidia of the former were 0–2-septate, whereas those of *Z. qianensis* were (0–) 1 (–7)-septate. The size of dark brown stipes of conidiophores of these two species also differed (Table S2). Furthermore, *Z. emperorae* and *Z. qianensis* formed completely different clades in the ITS phylogenetic tree. Therefore, *Z. emperorae* is considered as a new species of *Zygophiala* on the basis of its unique morphological characteristics and the ITS sequence analysis. We did not observe the teleomorph of *Z. emperorae*. The host range of *Z. emperorae* includes plants of Rosaceae: apple, pear, plum and pawpaw. This species is widespread in both northern and southern China.

**Figure 5:**
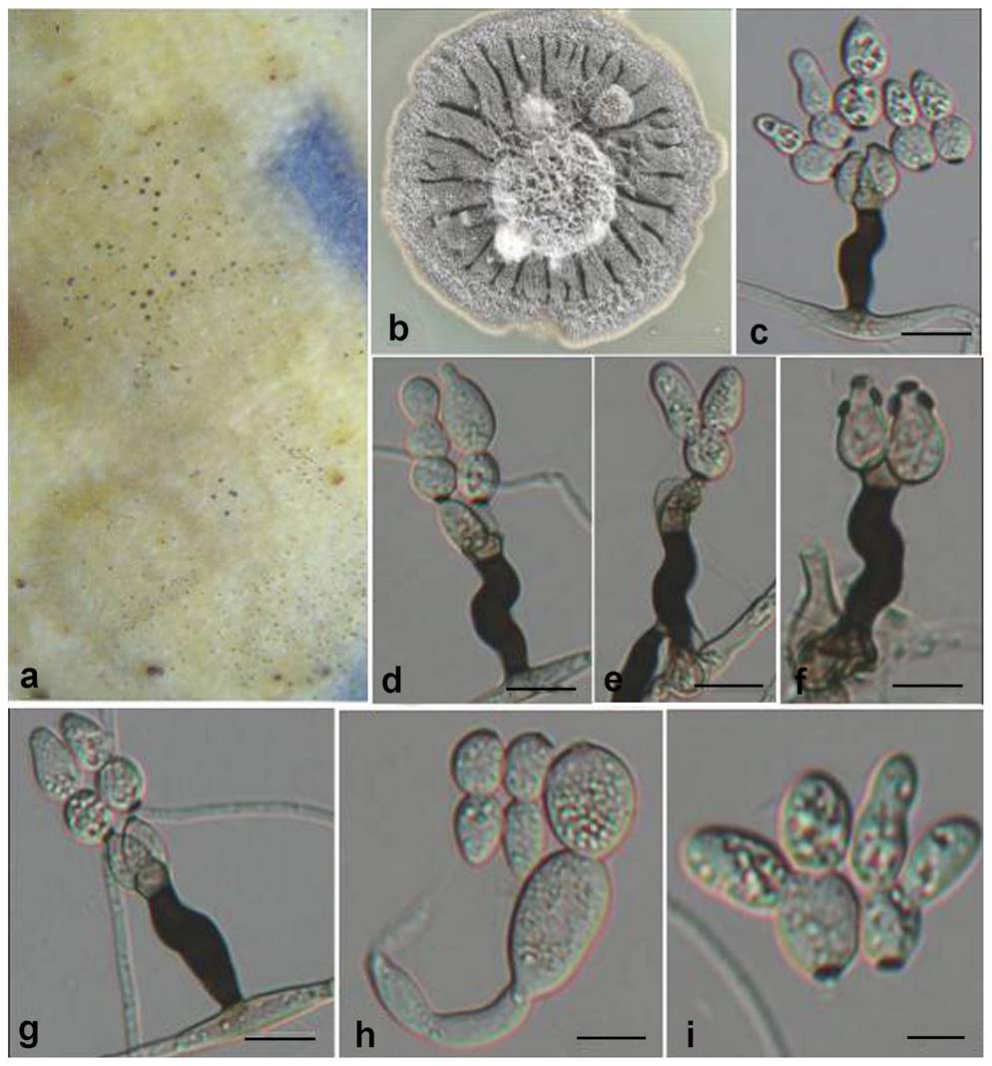
*Zygophiala trispora* isolate HL-HKBJ-23D. (a) flyspeck signs on banana peel (b) 1-month-old colony on PDA (c–i) conidiophores and conidia. bars c–g = 10 µm, h and i = 5 µm.

***Zygophiala trispora.*** G. Y. Sun & Liu Gao, sp. nov. (Fig. 5)

[urn:lsid:indexfungorum.org:names:808249]

#### Etymology

Named after the shape of its spores. Basal cell of conidia can give rise to two apical cells to form tricellular conidia.

*Conidiophores* arising from superficial hyphae, 2 µm wide, erect, scattered, 3- septate, subcylindrical, mostly flexuous, consisting of a hyaline to subhyaline supporting cell that gives rise to a smooth, dark brown stipe, 15–20×5–7 µm (from basal septum to below phialide), terminating in a finely verruculose, medium brown, angular apical cell, 4–5×3–4 µm, that gives rise to two light to medium brown, doliiform or elipsoidal, finely verruculose, polyblastic conidiogenous cells, 7–11×4–7 µm; scars prominent, darkened and thickened, apical and lateral, 2 µm wide. *Conidia* solitary, subcylindrical, or obclavate, hyaline, smooth and thick-walled, 1–2 septate; (10–) 15–20 (–25) × (4–) 5–7 (–8) µm, prominently constricted at septa; the apex long cylindrical, base subtruncate, basal cell of the conidia distinctly subcylindrical with a darkened, thickened hilum, 2 µm wide.

#### Cultural characteristics

Colonies dark olivaceous, slightly mounded, sometimes with small dark droplets in the center, margins corrugated or uneven, reaching 20 mm diameter after two weeks on OA on 25°C.

#### Holotypus

HMAS244987 ( =  HL-HKBJ-23D) (dried culture). From Japanese banana (*Musa basjoo* Siebold & Zucc.) fruit, Ledong, Hainan, China. Oct. 2010, L. Hao. Ex-type culture CGMCC3.17104 ( =  HL-HKBJ-23D).

#### Notes

We recognized *Z. trispora* as a new member of the genus *Zygophiala* based on morphological comparison and ITS sequence analysis. According to phylogenetic analysis, *Z. trispora* isolate HL-HKBJ-23D formed completely different clades in the ITS tree from other *Zygophiala* species. As for morphology, the basal cell of conidia can give rise to two apical cells, which presents a clover shape that is distinct from other species of *Zygophiala*. We did not observe the teleomorph of *Z. trispora.*

**Figure 6:**
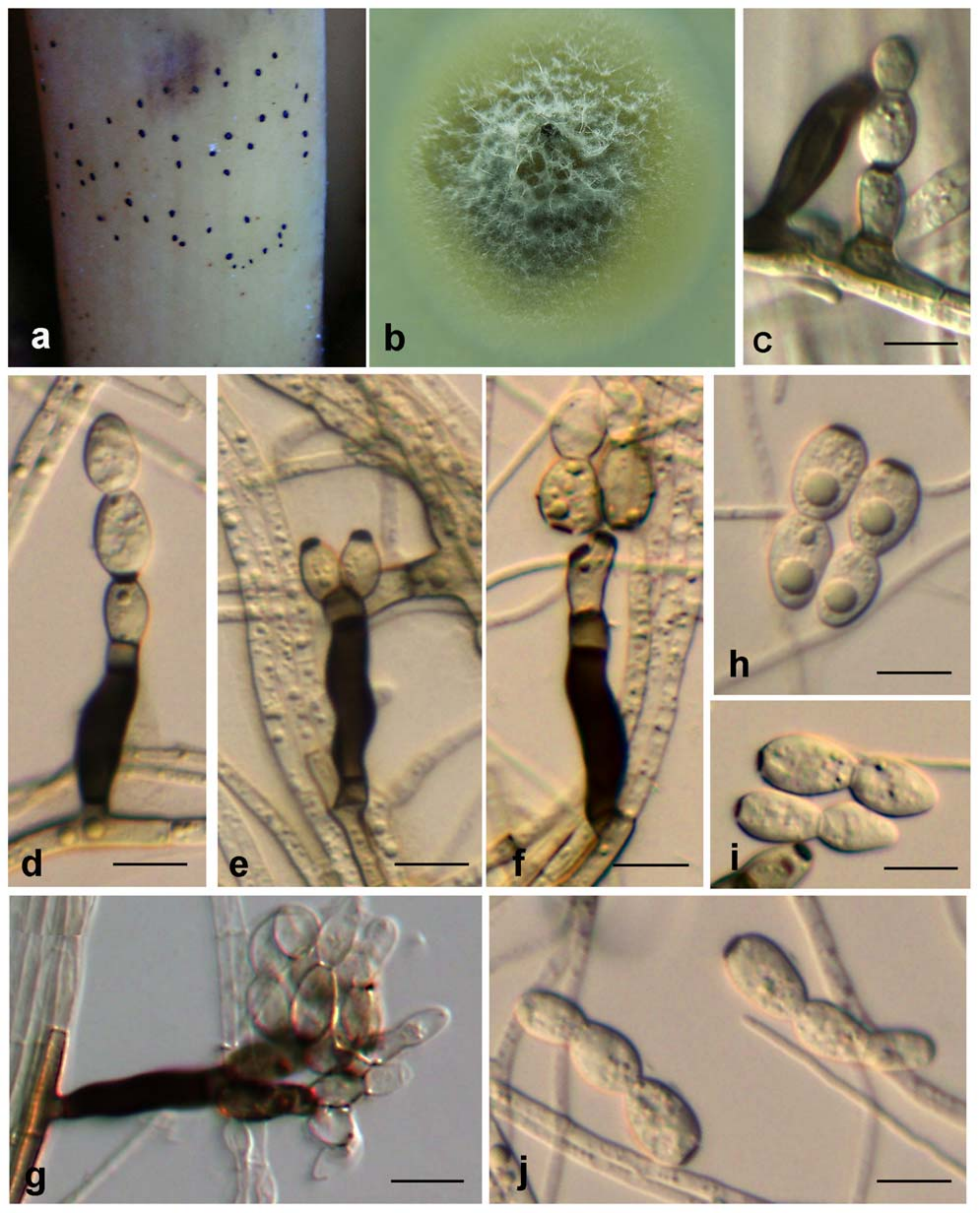
*Zygophiala musae* isolate CHC-HNBJ-2. (a) flyspeck signs on banana peel (b) 2-week-old colony on OA (c–g) conidiophores and conidia (h–j) conidia. bars  = 10 µm.

***Zygophiala musae.*** G. Y. Sun & Liu Gao, sp. nov. (Fig. 6)

[urn:lsid:indexfungorum.org:names: 808250]

#### Etymology

Named after the host, Japanese banana (*Musa basjoo* Siebold & Zucc), from which it was collected.

*Conidiophores* arising from superficial hyphae, 3–4 µm wide, erect, scattered, 3- septate, subcylindrical, irregularly bent, consisting of a hyaline to subhyaline supporting cell that gives rise to a smooth, dark brown stipe, 16–24×5–7 µm (from basal septum to below phialide), terminating in a finely verruculose, medium brown apical cell, 4–5×4–5 µm, that gives rise to one or two medium brown, finely verruculose, doliiform to ellipsoid or subcylindrical, polyblastic conidiogenous cells, 7–14×4–7 µm; scars prominent, apical and lateral, darkened, thickened, somewhat refractive, with 1–2 (–5) per conidiogenous cell, 2–4 µm wide. *Conidia* solitary, fusiform to obclavate, hyaline, smooth and thick-walled, transversely 1–2 septate, prominently constricted at septa, (17–) 21–26 (–30)×6–9 (–11) µm if 1-septate, 26–31×5–8 µm if 2-septate; apex subovoid, base subtruncate, the basal cell of the conidia subcylindrical, with a darkened, thickened hilum, 3–4 µm wide.

#### Cultural characteristics

Colonies on OA were olivaceous gray in the center, slightly mounded; margins, smooth, regular, aerial mycelium absent, faint yellow throughout, and reaching 14–15 mm diameter after 2 weeks at 25°C.

#### Holotypus

HMAS244989 ( =  CHC-HNBJ-2) (dried culture). From cuticle of Japanese banana (*Musa basjoo* Siebold & Zucc.) fruit, Ledong County, Hainan, China. Oct. 2010, H. C. Chen. Ex-type culture CGMCC3.15251 ( =  CHC-HNBJ-2).

#### Additional isolate examined

China, Chongqing, from plum (*Prunus salicina* Lindl.) fruit with flyspeck, Oct. 2010, H. C. Chen, CHCCQNL5.

#### Notes

The conidial size of *Z. musae* was relatively larger: (17–) 21–26 (–30)×6–9 (–11) µm if 1-septate, 26–31×5–8 µm if 2-septate. The size of the 1-septate conidia is similar to that of the anamorph of *S. pomi* and *Z. cylindrica*, and they are distinguishable by hilum size. Hila of *Z. musae* conidia were 3–4 µm wide, whereas those of *S. pomi* and *Z. cylindrica* were 2 and 2–3 µm wide, respectively (Table S2). In addition, the apex cells of conidia were somewhat pointed and subovoid in shape; this is morphologically distinct from other species of *Zygophiala*. We did not observe the teleomorph of *Z. musae.* Two isolates were obtained from different taxonomic classes of hosts - banana (Monocotyledons) and plum (Dicotyledons) - which suggested that *Z. musae* may have a wide host range.

**Figure 7:**
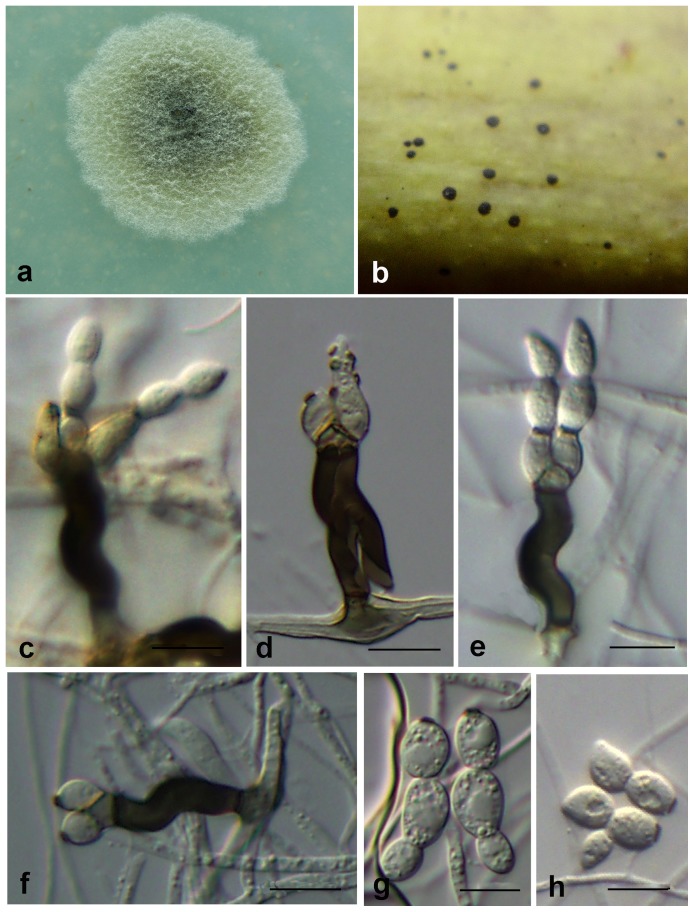
*Zygophiala inaequalis* isolate GL-MMXJ-52. (a) 2-week-old colony on OA (b) flyspeck signs on banana peel (c–f) conidiophores and conidia (g, h) conidia. bars  = 10 µm.

***Zygophiala inaequalis.*** G. Y. Sun & Liu Gao, sp. nov. (Fig. 7)

[urn:lsid:indexfungorum.org:names: 808251]

#### Etymology

Named after the shape of asymmetrical conidia.

*Conidiophores* arising from superficial hyphae, 1.5–2 µm wide, erect, scattered, 3-septate, subcylindrical, flexuous, consisting of a hyaline to subhyaline supporting cell that gives rise to a smooth, dark brown stipe, 17–22×4–6 µm (from basal septum to below phialide), terminating in a finely verruculose, medium brown, angular apical cell, 3–5×4–6 µm, that gives rise to two light to medium brown, finely verruculose, doliiform to ellipsoidal or elongated subcylindrical, polyblastic conidiogenous cells, 5–9×4–6 µm; scars prominent, apical and lateral, darkened, thickened, somewhat refractive, with 1 (–5) per conidiogenous cell, 2 µm wide. *Conidia* solitary, elongated subcylindrical, fusiform or obclavate, hyaline, smooth and thick-walled, transversely 1 (2)-septate, (11–) 13–16 (–20)×4–6 (–8) µm, strongly constricted at septa; apex ovoid, base subtruncate; with a darkened, thickened hilum, 2–3 µm wide.

#### Cultural characteristics

On oatmeal agar (OA), 2-week-old colonies spread with aerial mycelium with smooth, regular margins. The colour of the colony transitions from olivaceous gray in the center to pale white at the edge, reaching 20 mm diameter after 2-wk growth at 25°C.

#### Holotypus

HMAS244990 ( =  GL-MMXJ-52) (dried culture). From Japanese banana (*Musa basjoo* Siebold & Zucc.) fruit, Maoming, Guangdong, China. Oct. 2011, L. Gao, H. C. Chen & W. H. Li. Ex-type culture CGMCC3.15249 ( =  GL-MMXJ-52).

#### Additional isolate examined

China, Guangdong, Maoming, from Japanese banana (*Musa basjoo* Siebold & Zucc.) fruit, Oct. 2011, Oct. 2011, L. Gao, H. C. Chen & W. H. Li., GLMMXJ56A.

#### Notes

The apical cells of conidia were sometimes asymmetrical. The conidia of this species were the smallest among all other *Zygophiala* species, which is (11–) 13–16 (–20) ×4–6 (–8) µm. However, the hilum size of conidia was 2–3 µm, which was comparatively large among *Zygophiala* species (Table S2). The apex cells of conidia were ovoid, occasionally two apex cells grown out from one base cell. This is distinguishable in *Zygophiala.* We did not observe the teleomorph of *Z. inaequalis.* To date, this species has been isolated only from Japanese banana in southern China.

**Figure 8:**
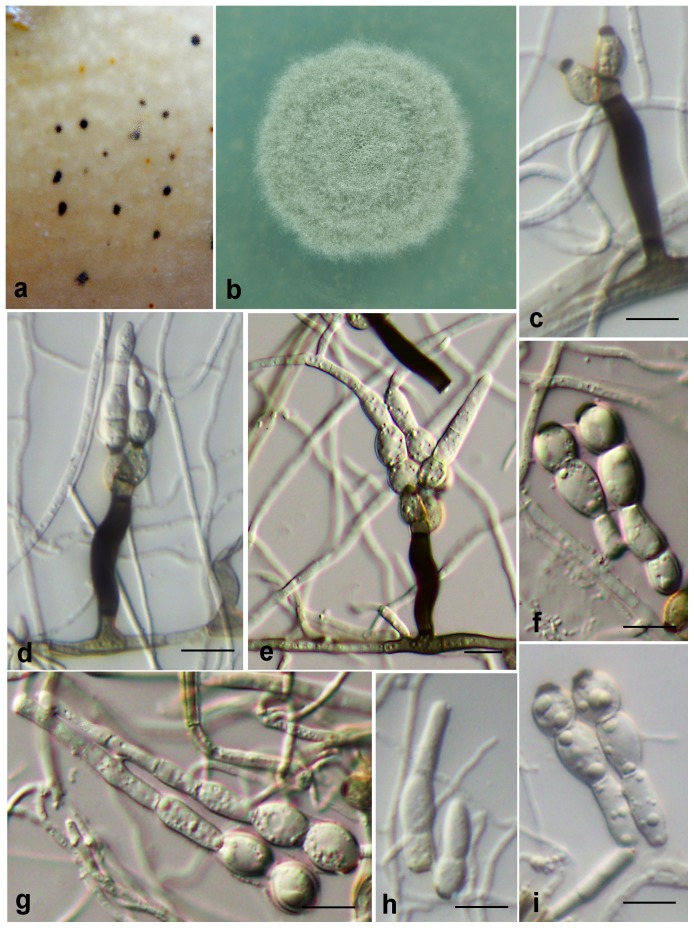
*Zygophiala longispora* isolate GL-GZXJ-39. (a) flyspeck signs on banana peel (b) 2-week-old colony on OA (c-e) conidiophores and conidia (f–i) conidia. bars  = 10 µm.

***Zygophiala longispora.*** G.Y. Sun & Liu Gao, sp. nov. (Fig. 8)

[urn:lsid:indexfungorum.org:names:808252]

#### Etymology

Named after the long conidium of this species.

*Conidiophores* arising from superficial hyphae, 2 µm wide, erect, scattered, 3-septate, subcylindrical, flexuous, consisting of a hyaline to subhyaline supporting cell that gives rise to a smooth, dark brown stipe, 26–3××6–8 µm (from basal septum to below phialide), terminating in a finely verruculose, medium brown, angular apical cell, 4–5×5–6 µm, that gives rise to two (rarely three) light to medium brown, finely verruculose, doliiform to ellipsoidal or elongated subcylindrical, polyblastic conidiogenous cells, 7–9×5–6 µm; scars prominent, apical and lateral, darkened, thickened, somewhat refractive, with one per conidiogenous cell, 2–3 µm wide. *Conidia* solitary, elongated subcylindrical, fusiform or obclavate, hyaline, smooth and thick-walled, transversely (1) 2–3 (–5)-septate, strongly constricted at septa; 1-septa, 17–20 (–22)×(5–) 6–9 µm; 2-septate, (25–) 28–32 (–34)×(6–) 7–9 µm; 3-septate, (27–) 40–46×(6–) 7–9 µm and up to 68 µm long if 5-septate; the apex cell subobtuse, sometimes clavate, base subtruncate, with a darkened, thickened hilum, 3–4 µm wide.

#### Cultural characteristics

On oatmeal agar (OA), 2-week-old colonies were spreading with aerial mycelium and smooth, regular margins. The colour of the colony was light gray, reaching 18 mm diameter after 2-week growth at 25°C.

#### Holotypus

HMAS244988 ( =  GL-GZXJ-39) (dried culture). From Japanese banana (*Musa basjoo* Siebold & Zucc.) fruit, Guangzhou, Guangdong, China. Oct. 2011, L. Gao, H. C. Chen & W. H. Li. Ex-type culture CGMCC3.15248 ( =  GL-GZXJ-39).

#### Notes

Most conidia of *Z. longispora* were 2 or 3-septate and were exceptionally long for this genus: (25–) 28–32 (–34)×(6–) 7–9 µm and (27–) 40–46×(6–) 7–9 µm, respectively. However, size of 1-septate conidia was smaller; only 17–20 (–22) × (5–) 6–9 µm, or smaller than those of the anamorph of *S. pomi* ((20–) 22–25 (–30) ×5–7 (–8) µm), *Z. cylindrica* ((17) 20–35 (41) × (4) 5–10 µm) or *Z. musae* ((17–) 21–26 (–30) ×6–9 (–11) µm). Nevertheless, the majority of conidia of *Z. longispora* averaged greater length than any other *Zygophiala* species. We did not observe the teleomorph of the *Z. longispora.*

***Zygophiala cryptogama*** Batzer & Crous, Mycologia 100: 254, 2008.

[urn:lsid:indexfungorum.org:names:501243]

#### Isolate examined

China, Gansu, Tianshui, from Red delicious apple (*Malus* × *domestica* Borkh.), Oct. 2011, L. Hao, HLMMQSG1.

#### Geographic distribution and host

This species was firstly described in the USA on apple fruit and it was also reported on wild plum (*Prunus americana*) in Iowa [36] and apple (*M.* × *domestica*) in northern China [13].

***Zygophiala wisconsinensis.*** Batzer & Crous, Mycologia 100: 255, 2008.

[urn:lsid:indexfungorum.org:names: 501245]

#### Isolates examined

China, Hunan, Zhangjiajie, from Japanese plum (*Prunus salicina* Lindl.) fruit, Oct. 2010, H. C. Chen; China, Hunan, Zhangjiajie, from Japanese plum (*Prunus salicina* Lindl.) fruit, Oct. 2010, CHCZJJNL17; China, Hunan, Zhangjiajie, from Japanese plum (*Prunus salicina* Lindl.) fruit, Oct. 2010, CHCZJJNL31; China, Yunnan, Dali, from persimmon (*Diospyros kaki* Thunb.) fruit, Oct. 2010, H. C. Chen, CHCDLSZ1; China, Shaanxi, Xianyang, from apple (*M.* × *domestica* Borkh.) fruit with flyspeck, Sep. 2007, Z. Zhang, ZZLQ63; China, Liaoning, Dalian, from apple (*M.* × *domestica* Borkh.) fruit, Sep. 2007, Z. Zhang, ZZDLSHH2; China, Hunan, Changsha, from Japanese plum (*Prunus salicina* Lindl.) fruit, Oct. 2010, L. Hao, HLCSPGL53; China, Hainan, from damson plum (*Prunus domestica* L.) fruit, Oct. 2010, L. Hao, HLHBLLZ29; China, Shaanxi, Xianyang, from apple (*M.* × *domestica* Borkh.) fruit with flyspeck, Sep. 2009, H. Y. Li, LHYTY2; China, Shaanxi, Xianyang, from apple (*M.* × *domestica* Borkh.) fruit with flyspeck, Sep. 2009, LHYTY3; China, Henan, Lingbao, from apple (*M.* × *domestica* Borkh.) fruit with flyspeck, Oct. 2009, H. Y. Li, LHYLB1; China, Henan, Lingbao, from apple (*M.* × *domestica* Borkh.) fruit with flyspeck, Oct. 2009, H. Y. Li, LHYLB6; China, Henan, Lingbao, from apple (*M.* × *domestica* Borkh.) fruit with flyspeck, Oct. 2009, H. Y. Li, LHYLB12; China, Henan, Lingbao, from apple (*M.* × *domestica* Borkh.) fruit with flyspeck, Oct. 2009, H. Y. Li, LHYLB14; China, Henan, Lingbao, from apple (*M.* × *domestica* Borkh.) fruit with flyspeck, Oct. 2009, H. Y. Li, LHYLB15; China, Hunan, Zhangjiajie, from *Malus* sp. fruit with flyspeck, Oct. 2010, C. Chen, CCZJJ69; China, Shaanxi, Shangluo, from hawthorn *(Crataegus pinnatifida* Bge.*)* fruit with flyspeck, Sep. 2010, C. Chen, CCSZSZ20.

#### Geographic distribution and host range

This species has a far-ranging geographic distribution and multiple hosts. It has been reported on apple fruit in the US [9], China [20], [36] and northeastern Turkey [11]. It also has been found on sweet persimmon fruit in Korea [37]. In this research, we encountered this species on apple, European plum, Japanese plum, persimmon, and hawthorn, and it was distributed in both temperate and tropical areas of China.

### Pathogenicity test

Five representative isolates of the new species of *Zygophiala* were selected for pathogenicity tests on apple fruit. After 10 wk of inoculation in the field, the species *Z. emperorae* (GL-QXLPPG-1) and *Z. longispora* (GL-GZXJ-39) presented flyspeck signs on apple fruit, and the signs were similar to those on the original host peels. The control apple fruit did not show any flyspeck signs. Inoculation in the incubator showed the same results as in the field. After re-isolating the sclerotium-like bodies and re-sequencing the isolates, we found that their ITS sequences were the same as the original ones. This result indicated that apple can be the host of *Z. longispora,* which was originally isolated from Japanese banana. The isolates of *Z. trispora* (HL-HKBJ-23D), *Z. musae* (CHC-HNBJ-2) and *Z. inaequalis* (GL-MMXJ-52), which were all isolated originally from peels of Japanese banana, did not produce any colonies on apple.

## Discussion

Before 2008, only one species, *Zygophiala jamaicensis,* had been described in the genus *Zygophiala.* “*Z. jamaicensis*” was reported on a very wide range of hosts, including 120 species in 44 families of seed plants [8], [16]–[18]. Recently, taxonomic classification and biogeographic research on the genus *Zygophiala* were conducted, focusing primarily on apple and its reservoir hosts [1], [3], [9], [11],[13],[14],[36],[38]. In this study, we investigated *Zygophiala* species in a wide range of hosts and regions in China. But there is no report of *Z. jamaicensis* from either our study or any of other recent research based on molecular data. Thus, we support the assertion of Batzer et al. [9] that, contrary to earlier published work, *Z. jamaicensis* is apparently not a widely distributed species. As some variations of *Z. jamaicensis* in culture morphology were observed on many hosts [16], [19], we speculate that many fungi originally identified as *Z. jamaicensis* may in fact have belonged to other *Zygophiala* species and unknown *Zygophiala* species remains to be discovered.

Gao et al. [12] described the process of formation of flyspeck signs by *Z. wisconsinensis*, which has clusters of shiny, black, round to ovoid, sclerotium-like bodies with no visible mycelial mat signs on the cuticle of hosts. They showed that sclerotium-like bodies typical of flyspeck were formed by hyphal knotting, and the primary sclerotium-like bodies ramified through intercalary hyphae to form secondary sclerotium-like bodies and eventually the intercalary hyphal networks dissolved, becoming invisible to the naked eye. We hypothesize that all 11 described *Zygophiala* species, including the six formerly presented species and our 5 new species, follow the same process of forming sclerotium-like bodies on the host as *Z. wisconsinensis*. This hypothesis deserves more testing in the future.

ITS sequence was used previously as a molecular basis for classification of *Zygophiala* [9], [13],[14]. In this study, a four-gene analysis incorporating ITS, TEF, TUB2 and ACT genes was used to discriminate species and verify phylogenetic relationships of *Zygophiala*. ITS sequence is useful for preliminary delineation of isolates of *Zygophiala*, but some confusion can occur when a large number of species are analyzed. For instance, *Z. trispora, Z. musae* and *Z. inaequalis* was well delimitated with high support when using only ITS sequences for analysis, but the lineage between *Z. emperorae*, *Z. wisconsinensis* and *Z. longispora* was weakly supported. When ITS-TEF-TUB2-ACT matrix sequences were used for analysis, these three species were grouped in different clades with strong support.

**Figure 9:**
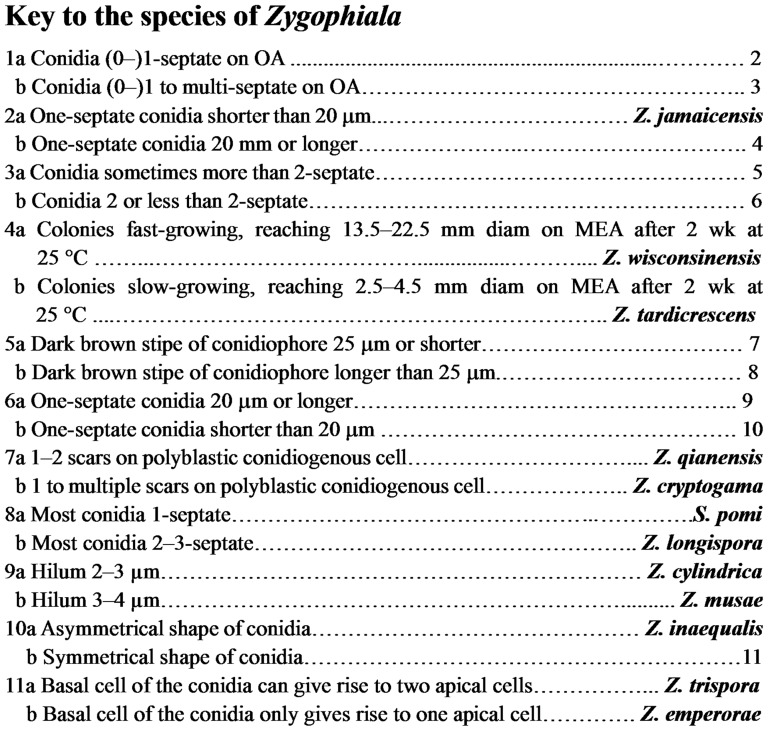
Key to the species of *Zygophiala*.

When this genus was first established, size of the conidiophore stipe, apical cell size, polyblastic conidiogenous cell size, septation, and conidia size were used to describe *Z. jamaicensis* [15]. Batzer et al. [9] used conidial size and number of septa to help distinguish between *Schizothyrium pomi* and *Z. cryptogama*, and rate of mycelial growth on malt extract culture media to differentiate between *Z. tardicrescens* and *Z. wisconsinensis*. We showed that additional characters, such as hilum size, size of the “S”-shaped conidiophore stipe, and number of scars on polyblastic conidiogenous cells could also be used to distinguish among *Zygophiala* species. For instance, *Z. qianensis* has 1–2 scars on polyblastic conidiogenous cell, whereas *Z. cryptogama* has 1 to multiple scars. *Z. cylindrica* and *Z. musae* can be distinguished by hilum size. The key to the species of *Zygophiala* was shown in Figure 9.

In recent literatures, host plants of newly described species only included apple, wild plum and sweet persimmon [9], [13], [14], [36], [38]. In this paper, the host range of *Zygophiala* was expanded to pear, hawthorn, persimmon, pawpaw and banana. Based on current evidence, hosts of *Z. wisconsinensis*, which was first isolated from apple fruit, are mainly Rosaceae plants, including apple, plum and hawthorn, but isolates of *Z. wisconsinensis* were also found on persimmon (Ebenaceae) (isolate CHCDLSZ1 in this paper and [38]). The hosts of *Z. emperorae* include apple, pear, plum and pawpaw. Japanese banana is also a host of *Zygophiala*; four of five new species in this paper were isolated from Japanese banana.

Pathogenicity tests on apple fruit indicated that the isolate of *Z. longispora* that originated from Japanese banana could infect apple both in the field and in an incubator, whereas other three new species of *Zygophiala* isolated from banana cannot. This preliminary evidence suggests that species of *Zygophiala* may differ with regard to host range.

Z. *wisconsinensis* is prevalent in many countries, including Korea [38], USA [6], [9], Turkey [11] and China [20]–[21]. Our survey data also suggest that Z. *wisconsinensis* is one of predominant species of *Zygophiala* in China, occurring in both southern and northern regions. *Z. emperorae* is another predominant species of *Zygophiala* in China, but it has not been found in other areas of the world. *Schizothyrium pomi* was not found in China although it is reported to be the most prevalent cause of flyspeck in the USA [6], [9] and in several European countries [6], [39]. Therefore, the worldwide geographic distribution patterns of *Zygophiala* species appear to be somewhat distinct depending on species.

## Supporting Information

Table S1 **Cultures of** ***Zygophiala*** **spp. used for morphological and molecular studies in species of Zygophiala.**
(DOCX)

Table S2 **Parameters used and statistical values resulting from the different phylogenetic analyses of individual datasets.**
(DOCX)

Table S3 **Conidiophore dimensions, septation and size of conidia.**
(DOCX)
