# Supplementary material for: Molecular and Morphological Analysis Reveals Five New Species of Zygophiala Associated with Flyspeck Signs on Plant Hosts from China
Source: PLoS One. 2014 Oct 20;9(10):e110717. doi: 10.1371/journal.pone.0110717 (PMC4203821; doi:10.1371/journal.pone.0110717)
Supplement: Table S1 — Cultures of Zygophiala spp. used for morphological and molecular studies in species of Zygophiala. (DOCX) [file pone.0110717.s001.docx]

**Table S1. Cultures of *Zygophiala* spp. used for morphological and molecular studies in species of *Zygophiala*.**

| **Species** | **Strain numbers** | **Host** | **Locality** | **ITS^c^** | **ACT^d^** | **TEF ^e^** | **TUB2^f^** |
| --- | --- | --- | --- | --- | --- | --- | --- |
| ***Schizothyrium pomi*** | **CUA1a, CBSa 118957** | ***Malus ×domestica*** | **USA** | **EF164898** | KJ578749 | KJ176961 | KJ578755 |
|  | **ZJ002** | ***Malus ×domestica*** | **USA** | **AY598849** | **–** | **–** | **–** |
|  | **AHA2a** | ***Malus* ×*domestica*** | **USA** | **AY598851** | **–** | **–** | **–** |
|  | PEA1a | *Malus* ×*domestica* | USA | KJ578752 | KJ578751 | KJ730239 | KJ578754 |
| ***Zygophiala cryptogama*** | **FVA2a, CBS^a^ 118949** | ***Malus* ×*domestica*** | **USA** | **AY598854** | **–** | **–** | **–** |
|  | **MWA8a** | ***Malus* ×*domestica*** | **USA** | **EF164899** | **–** | **–** | **–** |
|  | HLMMQSG1 | *Malus* sp. | Tianshui, Gansu | KF806029 | KJ176924 | KJ176948 | KJ176977 |
| ***Zygophiala wisconsinensis*** | **MSTA8a, CBS 118950** | ***Malus* ×*domestica*** | **USA** | **AY598853** | **–** | **–** | **–** |
|  | **GTA4b** | ***Malus* ×*domestica*** | **USA** | **AY598855** | **–** | **–** | **–** |
|  | CHCZJJNL17 | *Prunus salicina* | Zhangjiajie, Hunan | KF806018 | **–** | **–** | **–** |
|  | CHCZJJNL31 | *Prunus salicina* | Zhangjiajie, Hunan | KF806019 | **–** | **–** | **–** |
|  | CHCDLSZ1 | *Diospyros kaki* | Dali,Yunnan | KF806016 | **–** | **–** | **–** |
|  | ZZLQ63 | *Malus* ×*domestica* | Xianyang, Shaanxi | KF805991 | **–** | **–** | **–** |
|  | ZZDLSHH2 | *Malus* ×*domestica* | Dalian, Liaoning | KF805989 | **–** | **–** | **–** |
|  | HLCSPGL53 | *Prunus salicina* | Changsha, Hunan | KF806026 | KJ176925 | KJ176949 | KJ176978 |
|  | HLHBLLZ29 | *Prunus domestica* | Hainan | KF806027 | **–** | **–** | **–** |
|  | LHYTY2 | *Malus* ×*domestica* | Xianyang, Shaanxi | KF805994 | **–** | **–** | **–** |
|  | LHYTY3 | *Malus* ×*domestica* | Xianyang, Shaanxi | KF805995 | **–** | **–** | **–** |
|  | LHYLB1 | *Malus domestica* | Lingbao, Henan | KF805996 | **–** | **–** | **–** |
|  | LHYLB6 | *Malus* ×*domestica* | Lingbao, Henan | KF805997 | KJ176909 | KJ176935 | KJ176964 |
|  | LHYLB12 | *Malus* ×*domestica* | Lingbao, Henan | KF805998 | KJ176910 | KJ176936 | KJ176965 |
|  | LHYLB14 | *Malus* ×*domestica* | Lingbao, Henan | KF806020 | KJ176927 | KJ176951 | KJ176980 |
|  | **LHYLB15** | ***Malus* ×*domestica*** | **Lingbao, Henan** | **EU825775** | KJ176911 | KJ176937 | KJ176966 |
|  | CCZJJ69 | *Malus* sp. | Zhangjiajie, Hunan | KF857347 | KJ176912 | KJ176938 | KJ176967 |
|  | CCSZSZ20 | *Crataegus pinnatifida* | Shangluo, Shaanxi | KF806023 | KJ176913 | KJ176939 | KJ176968 |
|  | **ZXRBJ34** | ***Malus* ×*domestica*** | **Baoji, Shaanxi** | **EU329727** | **–** | **–** | **–** |
|  | **ZXRLN1** | ***Malus* ×*domestica*** | **Liaoning** | **EU329728** | **–** | **–** | **–** |
| ***Zygophiala tardicrescens*** | **MWA1a, CBS^a^ 118946** | ***Malus* ×*domestica*** | **USA** | **AY598856** | KJ578750 | KJ176960 | KJ578756 |
| ***Zygophiala cylindrica*** | **LHYLX3** | ***Malus* ×*domestica*** | **Baoji, Shaanxi** | **FJ941848** | **–** | **–** | **–** |
|  | **LHYYC9** | ***Malus* ×*domestica*** | **Yanan, Shaanxi** | **FJ941849** | **–** | **–** | **–** |
|  | ZMHS8 | *Malus* ×*domestica* | Republic of Montenegro | KF956056 | KJ176930 | KJ176957 | KJ176983 |
|  | ZMHS23 | *Malus* ×*domestica* | Republic of Montenegro | KF956057 | KJ176931 | KJ176958 | **–** |
|  | ZMHS33 | *Malus* ×*domestica* | Republic of Montenegro | KF956058 | KJ176932 | KJ176959 | **–** |
|  | ZMHS53 | *Malus* ×*domestica* | Republic of Montenegro | KF956059 | KJ176933 | KJ176956 | KJ176984 |
| ***Zygophiala qianensis*** | **MYQ-QXBS-06** | ***Malus* ×*domestica*** | **Xianyang, Shaanxi** | **FJ769236** | KJ176934 | KJ176955 | KJ176985 |
|  | MYNXY1.2 | *Malus* ×*domestica* | Xianyang, Shaanxi | KF806009 | **–** | KJ176953 | KJ176986 |
|  | MYNXY4.8 | *Malus* ×*domestica* | Xianyang, Shaanxi | KF806011 | KJ176929 | KJ176954 | KJ176982 |
|  | MYNPBXD2.5 | *Malus* ×*domestica* | Xianyang, Shaanxi | KF806010 | **–** | **–** | **–** |
|  | SJCW2 | *Malus* ×*domestica* | Xianyang, Shaanxi | KF806006 | **–** | **–** | **–** |
|  | HLMMGL2 | *Malus* sp. | Maoming, Guangdong | KF806028 | KJ176923 | KJ176947 | KJ176976 |
|  | LWHLNLZ14 | *Prunus domestica* | Liaoning | KF806030 | KJ176918 | KJ176944 | KJ176973 |
|  | ZZHL10 | *Malus* ×*domestica* | Yanan, Shaanxi | KF805992 | **–** | **–** | **–** |
| ***Zygophiala emperorae*** | GLQXLPPG1 | *Malus* ×*domestica* | Xianyang, Shaanxi | KF646710 | KJ176915 | KJ176941 | KJ176970 |
|  | GLQXLPPG2A | *Malus* ×*domestica* | Xianyang, Shaanxi | KF806024 | **–** | **–** | **–** |
|  | GLQXLPPG2 | *Malus* ×*domestica* | Xianyang, Shaanxi | KF806025 | KJ176916 | KJ176942 | KJ176971 |
|  | LHYGSN5 | *Malus* ×*domestica* | Gansu | KF805999 | **–** | **–** | **–** |
|  | LHYGSN6 | *Malus* ×*domestica* | Gansu | KF806000 | **–** | **–** | **–** |
|  | LHYBXTY1 | *Malus* ×*domestica* | Xianyang, Shaanxi | KF805993 | **–** | **–** | **–** |
|  | LHYBXDJG7 | *Malus* ×*domestica* | Xianyang, Shaanxi | KF806003 | **–** | **–** | **–** |
|  | LHYBXDJG8 | *Malus* ×*domestica* | Xianyang, Shaanxi | KF806004 | **–** | **–** | **–** |
|  | LHYLX2 | *Malus* ×*domestica* | Baoji, Shaanxi | KF806001 | **–** | **–** | **–** |
|  | SJLX1 | *Malus* ×*domestica* | Baoji, Shaanxi | KF806005 | **–** | **–** | **–** |
|  | SJBX2 | *Malus* ×*domestica* | Xianyang, Shaanxi | KF805985 | **–** | **–** | **–** |
|  | SJLX3 | *Malus* ×*domestica* | Baoji, Shaanxi | KF806007 | **–** | **–** | **–** |
|  | MYQLX01 | *Malus* ×*domestica* | Baoji , Shaanxi | KF806012 | **–** | **–** | **–** |
|  | MYQLX25 | *Malus* ×*domestica* | Baoji, Shaanxi | KF806013 | **–** | **–** | **–** |
|  | MYQLX29 | *Malus* ×*domestica* | Baoji, Shaanxi | KF806014 | **–** | **–** | **–** |
|  | CCWWZ2 | *Pyrus bretschneideri* | Liaoning | KF806031 | KJ176920 | KJ176962 | KJ176974 |
|  | CCZJJ10 | *Prunus salicina* | Zhangjiajie, Hunan | KF857349 | **–** | **–** | **–** |
|  | CCZJJ14 | *Prunus salicina* | Zhangjiajie, Hunan | KF857348 | KJ176914 | KJ176940 | KJ176969 |
|  | ZXRBJ14 | *Malus* ×*domestica* | Baoji, Shaanxi | KF806008 | **–** | **–** | **–** |
|  | ZXRBJ20 | *Malus* ×*domestica* | Baoji, Shaanxi | KF805986 | **–** | **–** | **–** |
|  | ZXRBJ31A | *Malus* ×*domestica* | Baoji, Shaanxi | KF805987 | **–** | **–** | **–** |
|  | ZXRBJ31B | *Malus* ×*domestica* | Baoji, Shaanxi | KF805988 | **–** | **–** | **–** |
|  | LYPYX12.1 | *Chaenomeles speciosa* | Yuxi, Yunnan | KF806022 | KJ176928 | KJ176952 | KJ176981 |
|  | ZZM7Y1 | *Malus* ×*domestica* | Baoji, Shaanxi | KF805990 | **–** | **–** | **–** |
| ***Zygophiala trispora*** | HL-HKBJ-23D | *Musa basjoo* | Haikou, Hainan | KF646711 | KJ176926 | KJ176950 | KJ176979 |
| ***Zygophiala musae*** | CHCCQNL5 | *Prunus salicina* | Chongqing | KF806017 | **–** | **–** | **–** |
|  | CHC-HNBJ-2 | *Musa basjoo* | Haikou, Hainan | KF646707 | KJ176922 | KJ176946 | KJ176975 |
| ***Zygophiala inaequalis*** | GL-MMXJ-52 | *Musa basjoo* | Maoming, Guangdong | KF646709 | KJ176921 | KJ176945 | KJ578753 |
|  | GLMMXJ56A | *Musa basjoo* | Maoming, Guangdong | KF806032 | KJ176917 | KJ176943 | KJ176972 |
| ***Zygophiala longspora*** | GL-GZXJ-39 | *Musa basjoo* | Guangzhou Guangdong | KF646708 | KJ176919 | KJ176963 | **–** |
| ***Teratosphaeria alistairii*** | **CPC^b^ 18251** | **–** | **–** | **JX556227** | **–** | **–** | **–** |

The isolates obtained from GenBank were showed in bold.

^a^CBS: CBS-KNAW Fungal Biodiversity Centre, Utrecht, The Netherlands.

^b^CPC: Culture collection of P.W. Crous, housed at CBS.

^c^ITS: Internal transcribed spacers 1 and 2 together with 5.8S nrDNA.

^d^ACT: actin gene.

^e^TEF: Translation elongation factor 1-alpha gene.

^f^TUB2: partial β-tubulin (tub2) gene.
