# Supplementary material for: Molecular and Morphological Analysis Reveals Five New Species of Zygophiala Associated with Flyspeck Signs on Plant Hosts from China
Source: PLoS One. 2014 Oct 20;9(10):e110717. doi: 10.1371/journal.pone.0110717 (PMC4203821; doi:10.1371/journal.pone.0110717)
Supplement: Table S2 — Parameters used and statistical values resulting from the different phylogenetic analyses of individual datasets. (DOCX) [file pone.0110717.s002.docx]

**Table S2.** **Parameters used and statistical values resulting from the different phylogenetic analyses of individual datasets.**

|  | **Dataset** | **ITS** | **TEF-1α** | **ACT** | **TUB2** | **Combined** |
| --- | --- | --- | --- | --- | --- | --- |
| **Number of characters** | **Total** | 487 | 251 | 279 | 400 | 1372 |
|  | **Variable** | 56 | 77 | 34 | 53 | 76 |
|  | **Constant** | 386 | 65 | 154 | 261 | 985 |
| **MP^a^** | **PIC^b^** | 45 | 109 | 91 | 86 | 311 |
|  | **Number of trees** | 100 | 52 | 100 | 2 | 6 |
|  | **Tree length** | 142 | 368 | 251 | 235 | 693 |
|  | **CI^c^** | 0.7887 | 0.7201 | 0.7570 | 0.7702 | 0.7244 |
|  | **HI^d^** | 0.2113 | 0.2799 | 0.2430 | 0.2298 | 0.2756 |
|  | **RI^e^** | 0.9367 | 0.8444 | 0.9039 | 0.9101 | 0.9009 |
|  | **RC^f^** | 0.7388 | 0.6081 | 0.6843 | 0.7010 | 0.6526 |
| **BI^g^** | **ASDSF^h^** | 0.008166 | 0.004968 | 0.006143 | 0.005825 | 0.003817 |

^a^MP: maximum parsimony.

^b^PIC: number of parsimony informative characters.

^c^CI: consistency index.

^d^HI: homoplasy index.

^e^RI: retention index.

^f^RC: rescaled consistency index.

^g^BI: Bayesian inference.
