# Supplementary material for: Molecular and Morphological Analysis Reveals Five New Species of Zygophiala Associated with Flyspeck Signs on Plant Hosts from China
Source: PLoS One. 2014 Oct 20;9(10):e110717. doi: 10.1371/journal.pone.0110717 (PMC4203821; doi:10.1371/journal.pone.0110717)
Supplement: Table S3 — Conidiophore dimensions, septation and size of conidia. (DOCX) [file pone.0110717.s003.docx]

**Table S3.** **Conidiophore dimensions, septation and size of conidia.**

| **Species** | **Conidiophore** | | | **Conidia** | | | **References** |
| --- | --- | --- | --- | --- | --- | --- | --- |
|  | **Dark brow stipe size of conidiophore (µm)** | **Apical cell size (µm)** | **Polyblastic conidiogenous cell size (µm)** | **Septation** | **Size of 1-septate conidia (µm)** | **Hilum size (µm)** |  |
| *Z. tardicrescens* | 14–16 × 5–6 | 3–4 × 4–6 | 7–10 × 5–6 | 1-septate | (12–) 14–18 (–20) × (4–) 5–6 (–8) | 2 µm | [9] |
| *Z. wisconsinensis* | 15–20 × 4–7 | 3–4 × 4–5 | 7–11 × 5–6 | 1-septate | (13–) 15–18 (–23) × (6–) 7–8 | 2–3 µm | [9] |
| *Z. cryptogama* | 17–22 × 4–5 | 3–4 × 4–5 | 6–15 × 5–6 | 1 (–2) -septate | (12–) 14–18 (–20) × (4–) 5–6 (–8) | 1–2 µm | [9] |
| *Z. jamaicensis* | 16–24 × 4–5 | 4–5 × 3–4 | 6–8 × 4 | 1-septate | 15–18 × 4–5 | – | [15] |
| *S. pomi* | 25–35 × 7–8 | 6–7 × 6–7 | 8–12 × 6–7 | 1 (–7)-septate | (20–) 22–25 (–30) × 5–7 (–8) | 2 µm | [9] |
| *Z. qianensis* | 13–26 × 4–7 | 3–5 × 4–6 | 4–9 × 3–6 | (0–) 1 (–7)-septate | 13–21 × 5–9 | 1.5–2 µm | [14] |
| *Z. cylindrica* | 17–31 × 4.5–9 | 3–8 × 3.5–7 | 4–8 (24) × 3–7 | (0) 1 (2)-septate | (17) 20–35 (41) × (4) 5–10 | 2–3 µm | [13] |
| *Z. emperorae* | 16–27 × 4–6 | 3–6 × 4–6 | 4–8 × 4–6 | 0–2-septate | 12–20 × 6–8 | 2 µm | This paper |
| *Z. trispora* | 15–20 × 4.5–7 | 4–5 × 3–4 | 7–11 × 4–7 | 1–2-septate; | 13–24 × 4.5–10 | 2 µm | This paper |
| *Z. musae* | 16–24 × 5–7 | 4–5 × 4–5 | 7–14 × 4–7 | 1–2-septate | (17–) 21–26 (–30) × 6–9 (–11) | 3–4 µm | This paper |
| *Z. inaequalis* | 17–22 × 4–6 | 3–5 × 4–6 | 5–9 × 4–6 | 1–2-septate | (11–) 13–16 (–20) × 4–6 (–8) | 2–3 µm | This paper |
| *Z. longisporum* | 26–30 × 6–8 | 4–5 × 5–6 | 7–9 × 5–6 | (1) 2–3 (–5)-septate | 17–20 (–22) × (5–) 6–9 | 3–4 µm | This paper |
